# Polymer-Based Biocompatible Packaging for Implantable Devices: Packaging Method, Materials, and Reliability Simulation

**DOI:** 10.3390/mi12091020

**Published:** 2021-08-27

**Authors:** Seonho Seok

**Affiliations:** Center for Nanoscience and Nanotechnology (C2N), University-Paris-Saclay, 91120 Palaiseau, France; seonho.seok@u-psud.fr

**Keywords:** biocompatible packaging, implantable, reliability, Finite element method (FEM), simulation

## Abstract

Polymer materials attract more and more interests for a biocompatible package of novel implantable medical devices. Medical implants need to be packaged in a biocompatible way to minimize FBR (Foreign Body Reaction) of the implant. One of the most advanced implantable devices is neural prosthesis device, which consists of polymeric neural electrode and silicon neural signal processing integrated circuit (IC). The overall neural interface system should be packaged in a biocompatible way to be implanted in a patient. The biocompatible packaging is being mainly achieved in two approaches; (1) polymer encapsulation of conventional package based on die attach, wire bond, solder bump, etc. (2) chip-level integrated interconnect, which integrates Si chip with metal thin film deposition through sacrificial release technique. The polymer encapsulation must cover different materials, creating a multitude of interface, which is of much importance in long-term reliability of the implanted biocompatible package. Another failure mode is bio-fluid penetration through the polymer encapsulation layer. To prevent bio-fluid leakage, a diffusion barrier is frequently added to the polymer packaging layer. Such a diffusion barrier is also used in polymer-based neural electrodes. This review paper presents the summary of biocompatible packaging techniques, packaging materials focusing on encapsulation polymer materials and diffusion barrier, and a FEM-based modeling and simulation to study the biocompatible package reliability.

## 1. Introduction

**Figure 1:**
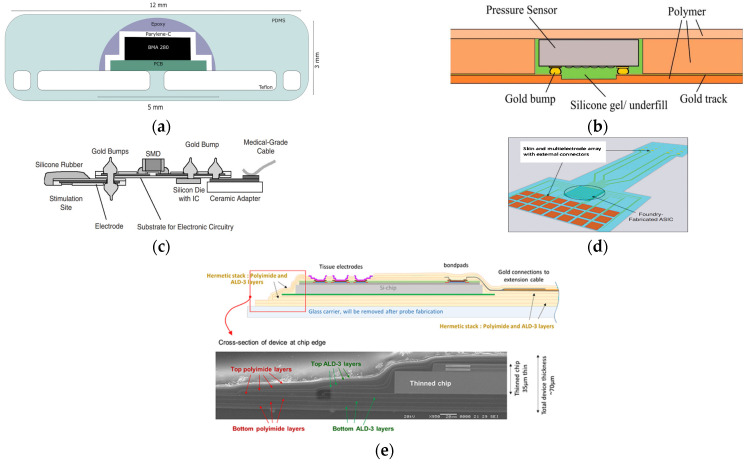
Examples of implantable device packaging. (**a**) PDMS encapsulation of wire-bonded chip; (**b**) Polyimide encapsulation of flip-chipped Si chip; (**c**) MicroFlex Interconnect (MFI); (**d**) MicroFlex Interconnect (MFI); (**e**) Ultra-Thin-Chip (UTC) packaging.

Evolution in IC (Integrated Circuit) packaging technology has been driven by the need for higher speed and higher density devices enabling smaller form factor and lower power consumption. For example, HBM (High Bandwidth Memory) has been developed by stacking memory die based on TSV (Through Silicon Vias) and stacking with micro-bump bonding in order to achieve higher bandwidth and thus lower power consumption [1,2,3,4]. Packaging of an implantable device is critical as it determines hermeticity and compatibility of the implant system in biological environment. Thus, reliability and life-time of the implant system highly depend on both packaging materials and technology. In general, implantable device packaging houses the electronic or mechanical system through polymer encapsulation [5,6,7,8,9,10,11,12], welding or bonding of metal [13,14], and ceramics [15]. Materials of the polymer encapsulation package include epoxies, silicones, polyurethanes, polyimides, silicon-polyimides, parylenes, polycyclic-olefins, silicon-carbons, benzocyclobutenes (BCB), and liquid crystal polymers. Conventionally, titanium (Ti) box has been used for packaging of an implantable electric device such as a pacemaker in order to ensure hermetic and biocompatible packaging of the microelectronic device. While it is a well-proven hermetic implant package, the Ti-box is rather large and rigid, which evokes a pronounced foreign body reaction (FBR) upon implantation, resulting in a thick fibrous tissue encapsulation, which might decrease the implant’s sensor sensitivity. Furthermore, mechanical mismatch of the Ti-box and local tissue might cause chronic discomfort for the patient [16]. Therefore, different packaging approaches have been reported to replace the existing Ti box package for implantable devices. Cardiac monitoring system has been implemented with commercial 3-axis accelerometer mounted on PCB as shown in Figure 1a. To be suitable for implant, parylene has been first coated on the surface of the accelerometer and PCB and then it is fixed on Teflon support providing stitching of the sensor on the cardiac wall with epoxy resin. Finally, a soft encapsulation in medical grade PDMS (NUSIL MED-6015) was fabricated around the device [17,18]. Pressure sensor device mounted on a stent graft has been flip-chip bonded to flexible, biocompatible polymer which has predefined metal feedthrough. After filling silicone gel around the bonded pressure sensor, a polymer has been laminated to seal the pressure sensor. A biocompatible silicone gel links the same with the thinned substrate layer in order to transfer the pressure within the aneurysm to the sensitive area of the sensor as shown in Figure 1b [19]. The flip-chip technology has been used to ensure miniaturization and flexibility of the device, compared to the commonly used wire bonding. Bare die assembly techniques such as flip-chip technology are the preferred choice, as they provide thin, small, and lightweight features and can be assembled on ceramic, laminate, Molded Interconnect Devices (MID) molded, and flexible substrates. Flip-chip technology can substitute and complement conventional surface-mounted devices (SMD) or wire bonding processes for an even higher degree of miniaturization [20]. An implantable retina stimulator is implemented by MFI (MicroFlex Interconnection) technology. The MFI technology is based on the common thermosonic ball-wedge bonding process. A gold ball is bonded by force, temperature, and ultrasound through the hole in the substrate on the IC pad as shown in Figure 1c. The metal pair is then welded together, resulting in mechanically and electrically stable interconnects [21,22]. Emergence of new implantable devices such as retina prothesis requires an innovative packaging as conventional wire-bonding techniques would not be applicable to implement massive electrical interconnect, for example, 1000 electrodes (see Figure 1d) [23]. In this case, technological barrier exists in substantial scale difference between Si chips and polymeric stimulation device for mechanical interconnection. As a solution, standard silicon wafer having through-holes of 2.51 × 2.63 mm^2^ is used as a temporary packaging platform. The 260-µm-thick chips are inserted from the backside and planarized using a tape on the front of the wafer. Photoresist sacrificial layer on the perimeter of the Si chip with help of anchoring parylene layer on the backside. From the frontside of the Si chip, parylene and metal interconnect has been fabricated to finalize the desired retina prothesis. The packaging and integration have been finished by separate the parylene with Si chips from the temporary Si platform through photoresist sacrificial etch [23]. Emergence of UTC (Ultra Thin Chip) opens new pathway of miniaturization of biocompatible package causing minimal neural tissue damage upon implantation for neural electrodes as shown in Figure 1e [24,25]. The initiation of UTC is made by need for embedding Si chip into packaging carrier substrate, which could reduce packaging cost by suppressing certain step of conventional packaging, for example, EMC (Epoxy Mold Compound) for flip-chip technology.

From the examples shown in Figure 1, it is found that the biocompatible packaging starts to use conventional packaging technology such as wire-bonding, flip-chip bonding, PCB chip carrier and advances to use microfabrication technology due to the need for maximum miniaturization and lots of electrical wiring. Implantable microsensors have been packaged in a conventional method such as EMC (Epoxy Mold Compound), wire bonding, PCB, and they are bonded and encapsulated with soft or biocompatible material to be suitable in biological environment. Furthermore, advanced medical device such as neural prosthesis is implemented with flexible material, its electrical interconnection with silicon IC is the essence of packaging technology. Thinned Si IC can be embedded into polymer material and it can be integrated with the polymer-based neural prothesis. Such a thin silicon chip is ideally best approach to achieve maximum miniaturization of an active implant. This paper presents polymer-based biocompatible packaging techniques for implantable devices. Biocompatible packaging approaches, focusing on materials and the packaging process, have been summarized in Section 2. Section 3 addresses the reliability issues of the biocompatible packaging as well as FEM modeling and simulation based on interfacial fracture mechanics.

## 2. Biocompatible Packaging Methods

The objective of the biocompatible packaging is to provide a protection for the implanted electronic device to be tolerating the harsh biological environment in order to increase life-time of implanted device. Sealing of the implanted device is one of the critical aspects of long-term reliable biocompatible package. Materials and process will determine the type of sealing of the biocompatible packaging; hermetic, watertight, or permeable. Particular difficulty of the biocompatible packaging is the need for feedthrough as the implanted device should interact with the biological medium in different ways; electrically, chemically, mechanically, or optically. It is required that the feedthrough should withstand mechanical stress due to biological environment such as muscle activity. In addition, it should not add significantly to the mechanical load of the implant, as in the case of tethered neural implants whose cable weight and flexibility may negatively affect tissue response [26,27]. As shown in Figure 1, it can be said that the biocompatible packaging is mainly being achieved in two different ways: (1) polymer encapsulation of conventional circuit board (2) chip-level packaging.

### 2.1. Polymer Encapsulation

**Figure 2:**
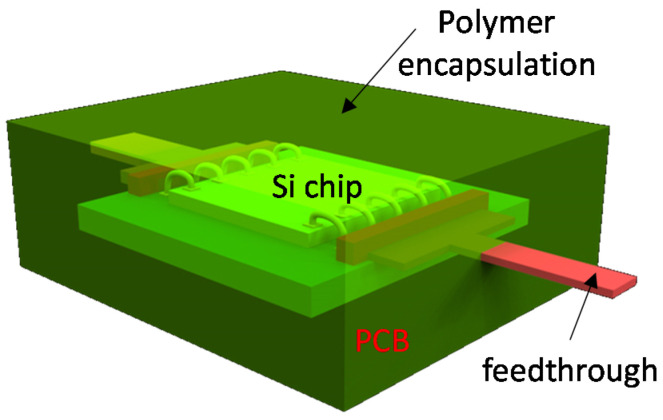
Polymer encapsulation of conventional package for biocompatible package.

Figure 2 shows conceptual drawing of polymer encapsulation of Si chip wire-bonded on PCB board. The role of polymer encapsulation is to protect the packaged circuit preventing biofluid from penetrating during its operation. Such a polymer encapsulation is implemented through a molding process which uses predefined mold to encapsulate the implanted electronics. To be well-bonded and guaranteed their mechanical properties, the polymer materials should be cured at designated temperature for suitable time duration. It could make additional thermal stress on the implantable device in conventional package and sometimes newly-formed interfaces between encapsulated device and encapsulation polymer suffers from delamination. In addition, this packaging approach has drawbacks in view of miniaturization of implantable devices as it houses conventional package with biocompatible polymer encapsulation. Most of the biocompatible packaging uses this approach even for advanced implantable neural devices. Material properties and process conditions of frequently-used polymer materials are summarized in Table 1 [28]. Polymer material has lower mechanical modulus, which reduces mechanical stress to surrounding tissues. The polymer materials are utilized through coating for molding or bonding for lamination to encapsulate the package inside.

Most of the polymer materials need to be cured at certain temperature in order to ensure stable mechanical properties. For example, epoxy will have different mechanical properties depending on curing conditions. Epoxy, cured at room temperature for 24 h (equivalently around 50% curing rate), shows yield strength of 24 MPa and elastic modulus of 2 GPa, while it has 35 MPa yield strength and 5 GPa Young’s modulus after 4 h curing at 64 °C [29]. In addition, hydrophobic surface treatment of the polymer is one of good solutions to improve the reliability of the biocompatible package [30,31,32].

### 2.2. Chip-Level Packaging

The important perspectives in implantable electronic package are biocompatibility, hermeticity, and miniaturization. Biocompatibility typically refers to the way the body tolerates the presence of an implant material and thus polymer materials given in Table 1 and Ti (titanium) box are frequently used for the biocompatible package as explained in previous section Hermeticity of a package is referred as the integrity of sealed packages to resist gas and liquids penetrating the seal or an opening (crack) in the package, especially critical to the reliability and longevity of a packaged electronics. A diffusion barrier based on thin-film passivation can be deposited onto a Si chip or a polymer encapsulation in order to prevent biofluid penetration or diffusion of IC materials into the body. Table 2 summarizes material properties of frequently-used diffusion barrier of the biocompatible package. Diffusion barrier is a dielectric film deposited on a Si surface or an encapsulating polymer to avoid liquid passage through biocompatible package [33,34,35,36,37,38,39,40]. The dielectric layer, Al_2_O_3_ deposited by ALD (Atomic Layer Deposition) is frequently used as moisture barrier between polymer layers to increase adhesion strength between the polymer layers or enhance the life-time of a neural electrode bilayered with parylene material [41,42]. Ultra-thin thermally-grown silicon dioxide transferred to flexible substrate has shown high robustness as biofluid barrier compared with conventional approaches such as LCP and Al_2_O_3_/Parylene-C [43].

**Figure 3:**
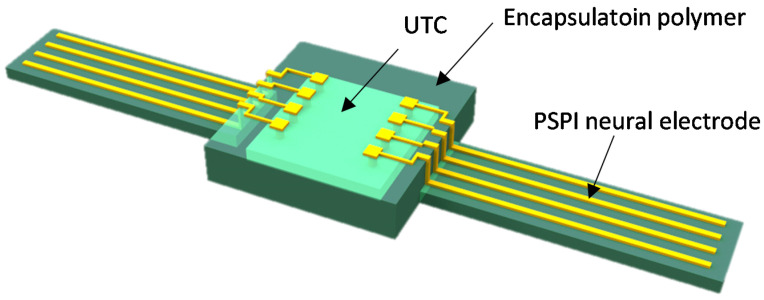
Conceptual drawing of chip-scale biocompatible package.

Figure 3 shows a concept of UTC-based chip-scale biocompatible package. It has merits of miniaturization compared with polymer encapsulation of conventional package. Miniaturization of the biocompatible package is important because mechanical load of the implanted device may negatively affect tissue response. Therefore, chip-scale biocompatible packaging may be more suitable for advanced implantable devices compared to the previous polymer encapsulation. It is realized, with microfabrication technologies, combining Si chips and polymer-based devices such as neural probe, retina prostheses, etc. It would make it possible to integrate Si chip into soft materials for neural probes without utilizing a chip carrier such as PCB. Technological difficulties of the integration process with two different materials rise in size mismatch between Si chips and polymer devices and thus innovative integration techniques, including metallization, are highly demanded. The Si chips are typically in a range of 200 µm after fab-out, while the polymer devices have total thickness up to tens of micrometers even if multiple polymer layers have been used. In general, typical Si chip having a few hundred micrometers in thickness is assembled to flexible circuit through flip-chip bonding with metallic bumps such as copper, gold, etc. Anisotropic Conductive Adhesive (ACA) is a good approach to integrate Si chips onto flexible substrate [44,45,46]. The advantages of ACA are reduced processing steps, lower processing temperature, and fine pitch capability. Stability of ACA’s electrical or mechanical performance depends on associated adhesive types; thermoplastics, such as polymer, or thermosetting, such as epoxies and silicones. ACA flip-chip technology is used to assemble bare chips where the pitch is extremely fine, normally less than 120 µm. ACA flip-chip bonding exhibits better reliability on flexible chip carriers because the ability of flex provides compliance to relieve stresses. For example, the internal stress generated during resin curing can be absorbed by the deformation of the chip carrier. ACA joint stress analysis indicated that the residual stress is larger on rigid substrates than on flexible substrates after bonding [47]. The thickness of ACA has ranged from 20 µm to 75 µm depending on the associated materials, and the process temperature is usually less than 200 °C. The drawback of ACA technique exists in that it needs a bonding process that is a reason of low throughput, and the finest pitch it can provide is limited down to hundred micrometers, as mentioned earlier. In case of retina protheses, it requires a great of number of interconnects for 1000 electrodes [48]. The packaging methods relying on bonding would not be desirable due to its low throughput and thus an innovative way of integration is necessary to achieve high density (fine pitch) chip-scale integrated interconnect packaging. Standard microfabrication-based chip-level packaging enables the density of interconnects to scale to the limits of photolithography used to define the etch holes over the on-chip pads [48]. However, integration packaging process is not simple because it is based on sacrificial layer release of the temporary guiding substrate. The guide substrate is required to hold relatively thicker Si chips compared with the parylene-based neural interface device during the microfabrication interconnect process. Such non-conventional processes sometimes create process errors such as misalignment between pad on Si chip and neural electrode, which eventually deteriorates the process yield as well as process cost. Ultra-thin-chip (UTC), defined as less than 20 µm in thickness, packaging could be a solution to tackle the process barrier related with thickness mismatch between Si chip and polymer materials. Fabrication of UTC is a big challenge and it can be implemented in different ways; grinding, epitaxial growth, SOI, silicon wafer with buried cavities, etc. Furthermore, UTC having 10–50 µm thickness shows good flexibility and good mechanical stability and it provides excellent flexibility and unconditional stability when its thickness is less than 10 µm. Embedded UTC in a polymer encapsulation may have great advantage because it provides low mechanical stress as well as biocompatibility. Such a thin Si chips may also be beneficial in view of process compatibility between polymer materials and silicon.

## 3. Reliability of Biocompatible Package

The biocompatible packages should be tested to estimate the reliability and life time as conventional packages. It is generally carried out through an accelerated aging test, which uses aggravated environmental conditions, such as temperature and humidity, to predict the expected life time of test devices or packages. In electronic package, the flip-chipped Si IC must use organic underfill to protect solder bumps by substantially reducing the mechanical stress. However, the underfill may create a new failure mode of the package due to CTE (Coefficient of Thermal Expansion) mismatch between the materials in joint. Shear or peeling stress could result in delamination of imperfect underfill with voids or microcracks under temperature cycling conditions. Such delamination is considered as mixed mode interfacial fracture and thus it is studied using FEM modeling and simulation [49,50,51,52,53]. Concerning biocompatible package, it is also similar case to the underfill of flip-chipped Si chip because polymer encapsulation creates multiple interfaces with the encapsulated Si chips, as explained earlier. Therefore, the theory of interfacial fracture mechanics is briefly explained, and then, an example of FEM modeling and simulation based on fracture mechanics is presented.

### 3.1. Estimation of Package Life-Time through Acceleration Aging Test

The acceleration aging test simulates real-time aging using elevated temperatures to artificially speed up the aging process. This test enables to get the expected life-time of the device under test. The estimation of the life-time can be calculated by using Arrhenius equation as follows [54].

(1) $$k=Ae^{\frac{-E_{a}}{k_{B}T}}$$

where *k* is rate constant, *A* is constant, *E_a_* is the activation energy, *k_B_* is the Boltzmann constant and *T* is absolute temperature.

If an acceleration aging test is running at *T*_2_ instead of *T*_1_, destruction will occur at a rate *k*_2_ where

(2) $$\log\frac{k_{2}}{k_{1}}=\frac{E_{a}}{k_{B}}\left(\frac{1}{T_{1}}-\frac{1}{T_{2}}\right)$$

Suppose rate doubles between 32 °C and 42 °C,

(3) $$\log2=\frac{E_{a}}{k_{B}}\left(\frac{1}{305\;{}^{\circ}K}-\frac{1}{315\;{}^{\circ}K}\right)$$

Therefore, $\frac{E_{a}}{k_{B}}=6663\;{}^{\circ}K\;\left(Hence\;E=0.58\;\mathrm{eV}\right).$ Suppose the test temperature is 67 °C, whereas the temperature in life is 37 °C.

(4) $$\log\frac{k_{2}}{k_{1}}=6663\left(\frac{1}{310\;{}^{\circ}K}-\frac{1}{340\;{}^{\circ}K}\right)$$

Therefore, $\frac{k_{2}}{k_{1}}=6.66$, the speed up factor is achieved.

Therefore, the test period for a 5-year life should be $\frac{5\;years}{6.66}=274\;days$.

### 3.2. Interfacial Fracture Mechanics

**Figure 4:**
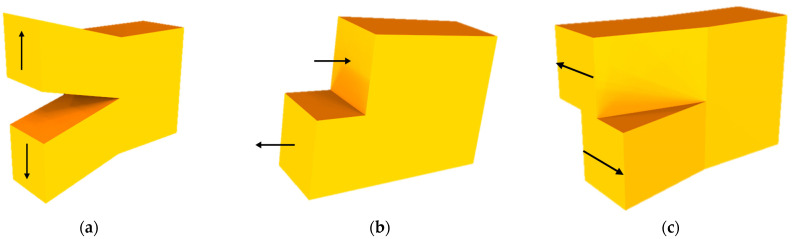
Basic fracture modes. (**a**) mode I (opening); (**b**) mode II (shearing); (**c**) mode III (tearing).

Conventional crack has been dealt in assumption that the material is homogeneous, but packaging for electronics or implantable device should be considered as non-homogeneous materials, creating different interfaces. In fracture mechanics, there are three types of cracks, referred to as mode I, II, and III, as shown in Figure 4. Mode I is a normal opening mode, while mode II and III are shear sliding mode and shear tearing mode, respectively. In case of homogeneous material, any fracture mode may be described by one of the three basic modes or their combination. The stresses near crack tip in the crack plane (xz-plane) for these three modes can be expressed as (*y* = 0, *x*->0+) [55],

(5) $$\sigma_{yy}=\frac{K_{I}}{\sqrt{2\pi x}}+O\left(\sqrt{x}\right),\;\sigma_{xy}=\sigma_{yz}=0$$

(6) $$\sigma_{xy}=\frac{K_{II}}{\sqrt{2\pi x}}+O\left(\sqrt{x}\right),\;\sigma_{yy}=\sigma_{yz}=0$$

(7) $$\sigma_{yz}=\frac{K_{III}}{\sqrt{2\pi x}}+O\left(\sqrt{x}\right),\;\sigma_{yy}=\sigma_{xy}=0$$

respectively, where the three parameters *K_I_*, *K_II_*, and *K_III_* are named stress intensity factors corresponding to the opening, sliding, and tearing (anti-plane shearing) modes of fracture, respectively. These equations shows that stress tend to be infinity as *x* approaches to crack tip.

Bimaterial interfacial cracks tend to exhibit mode mixity with coupling between mode I and mode II [54]. Stress along the interface ahead of crack tip is given

(8) $$\sigma_{yy}+i\sigma_{xy}=\frac{Kx^{i\varepsilon }}{\sqrt{2\pi x}}$$

where $K=K_{I}+iK_{II}$, x is the distance from crack tip, $\sigma_{yy}$ and $\sigma_{xy}$ are stress component normal and parallel to crack surface, respectively. The oscillatory index, *ε*, a function of the material properties

(9) ε=12πlnκ1μ1+1μ2κ2μ2+1μ1

where *µ* is shear modulus, *κ* = 3–4 *ν* for plane strain or = (3 − *ν*)/(1 + *ν*) for plane stress. The subscripts 1 and 2 represent each material associated to build the interface.

Phase angle, a measure of mode mixity, is given as follows.

(10) $$\psi =tan^{-1}\left(\frac{K_{II}}{K_{I}}\right)$$

Another important parameter for interface fracture, energy release rate *G*, can be found

(11) $$G=\frac{1}{cosh^{2}\left(\pi \varepsilon \right)}\frac{{\left|K\right|}^{2}}{E^{*}}$$

where $\frac{2}{E^{*}}=\frac{1}{E_{1}}+\frac{1}{E_{2}}$

The delamination will occur when the stress energy release rate *G* falls in following condition

(12) $$G\;\geq G_{c}\left(\psi \right)$$

where *Gc* is critical stress release rate of an interface, which is determined through experimental characterization.

### 3.3. FEM Simulation of a Biocompatible Package

**Figure 5:**
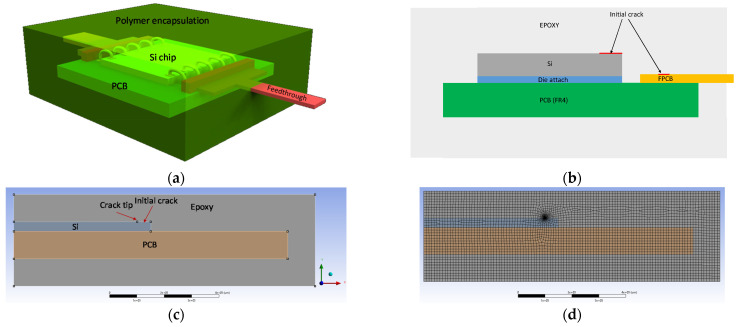
Finite element method (FEM) model of the biocompatible package. (**a**) Concept; (**b**) Cross-sectional view; (**c**) 2D model; (**d**) Meshed model.

**Figure 6:**
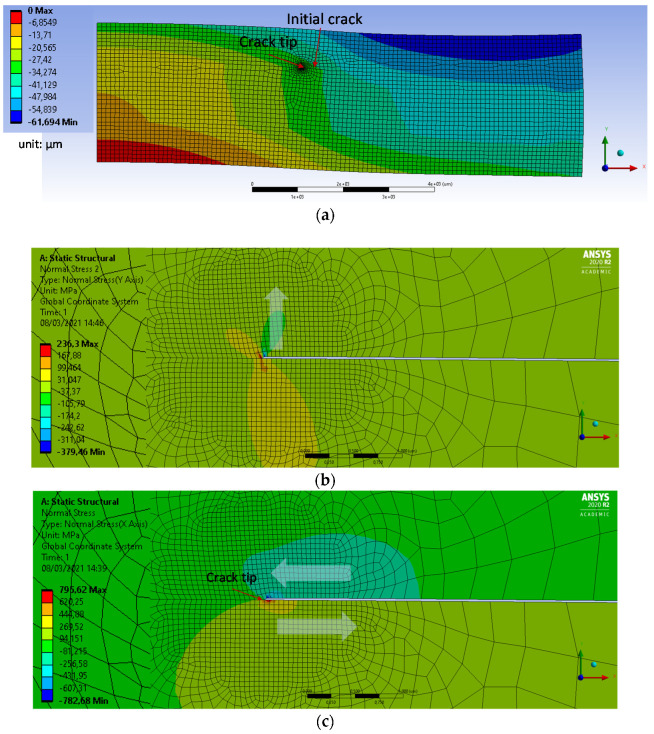
Package deformation and corresponding fracture mode at crack tip. (**a**) Deformation of the package; (**b**) Opening mode at crack tip; (**c**) Shear mode at crack tip.

Finite element method (FEM) simulation, with appropriate mechanics theories, becomes useful to find a solution of the reliability issues of different package structures. For example, underfill has significant impact on flip chip package reliability due to delamination driven by coefficient of thermal expansion (CTE) mismatch between organic substrate and silicon die. Such a delamination problem can be solved through FEM study of the effects of various design variables, including underfill material properties, fillet dimensions, and die overhang on underfill delamination fracture parameters. Since the delamination can be considered as a bimaterial interfacial crack, the FEM study is based on interfacial fracture mechanics, fundamentally a mixed mode, including both energy release rate and phase angle [38]. Likewise, the biocompatible package can be simulated through FEM modeling to study its reliability issues concerning delamination of encapsulating polymer. Figure 5a shows a conceptual drawing of biocompatible package for FEM simulation. The Si chip is attached to PCB carrier with glue and then wire-bonded for electrical connection. Flexible cables are connected with typical flexible cable connector at I/O ports of the Si chip. The conventional package has been encapsulated with a biocompatible polymer. Failure of such biocompatible package could be caused by imperfect encapsulation material with voids or microcracks as indicated in Figure 5b. Therefore, the failure mode, due to the initial crack, is of interest for the FEM simulation. Figure 5c presents 2D FEM model for fracture analysis due to the initial crack. The initial crack has been defined at the interface between Si chip and encapsulation polymer, epoxy. As it is a half model of the package, boundary condition has been correspondingly defined at symmetric line; ux = uy = 0 at x = y = 0, ux = 0 at x = 0. Table 3 summaries material properties and dimension of the model. The 2D element behavior is defined as axisymmetric or plane strain and applied temperature load is −100 °C in assumption that the package is under the acceleration aging test for its life-time estimation. Initial crack with crack tip is defined starting from Si edge at the interface with encapsulation epoxy. As the objective of this simulation is to find stress parameters related with the failure mode, finer mesh has been defined at the crack tip as shown in Figure 5d. The applied thermal load from the acceleration aging test makes the biocompatible package deformed due to CTE difference between materials. Thus, the deformation of the package has been first found in the simulation as shown in Figure 6a. The package has deformation of out-of-plane bending due to the thermal loading and thus Si chip is under tensile stress as expected.

Next, the crack modes at the crack tip have been checked. Figure 6b,c clearly demonstrates the direction of opening and shearing modes of an initially closed crack interface. It is important to understand the change of fracture parameters, including both energy release rate and phase angle, during crack propagation, i.e., increase in crack length.

**Figure 7:**
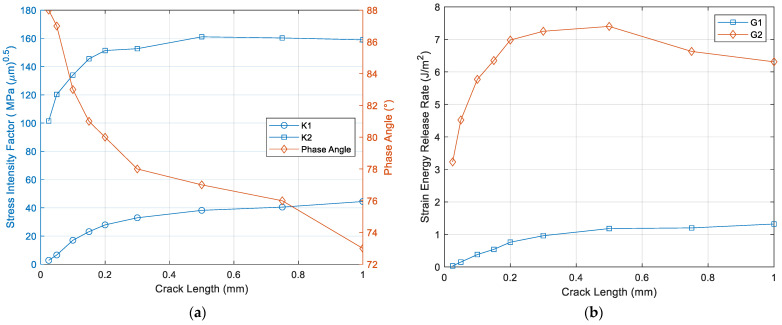
(**a**) Stress intensity factor, phase angle as function of crack length, and (**b**) strain energy release rate as function of crack length.

Figure 7 shows stress intensity factor, phase angle, and strain energy release rate as function of crack length, respectively. Opening mode (K_I_) and shear mode (K_II_) increase as the crack length increases. After 0.5 mm crack length, K_II_ is slightly reduced and K_I_ is still increasing, which is confirmed in phase angle change as function of crack length. Phase angle starts from near 90° (shear mode) and shows 72° at 1 mm crack length. Phase angle of 0° represents opening mode. Therefore, shear mode strength of epoxy is important when crack length is small, while tensile mode strength becomes important when crack length is substantial. SERR in mode 2 (G_2_) has maximum at 0.5 mm crack length, while SERR in mode 1 (G_1_) increases in the crack length of interest.

In case of small crack length, mode 2 is a dominant factor, as validated with phase angle and thus, critical toughness (G_c_) in mode 2 becomes important. When the crack length becomes substantial, critical toughness (G_c_) in mode 1 plays an important role. In general, G_c_ in mode 2 has bigger value that that of in mode 1 [53].

**Figure 8:**
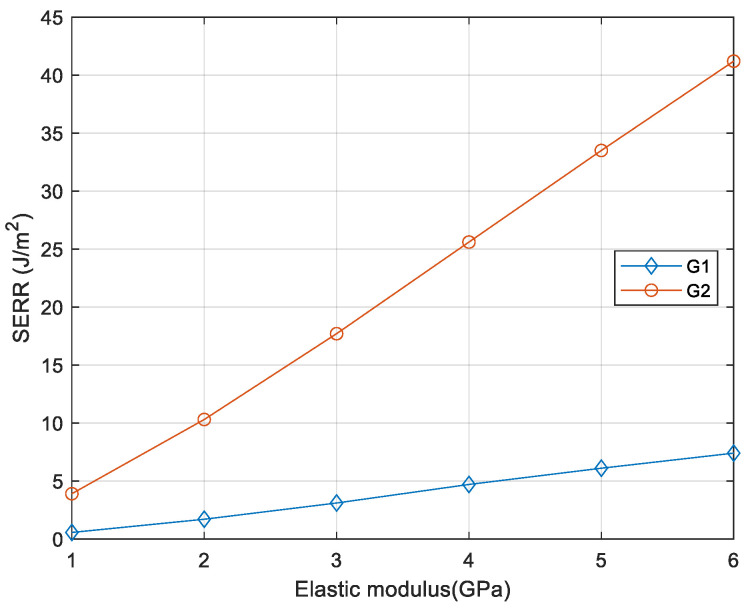
Strain energy release rate (SERR) as function of elasticity of packaging polymer.

The effect of material property of the packaging material has been studied as it is one of major critical parameters. The encapsulation polymer is the packaging material for biocompatibility, so its elasticity has been changed to check its effect to the packaging reliability. As seen in Figure 8, strain energy release rate (SERR) increases with elastic modulus of encapsulation material, which indicates that stiffer encapsulation will generate a larger crack driving force and accelerate crack propagation once the crack is initiated.

**Figure 9:**
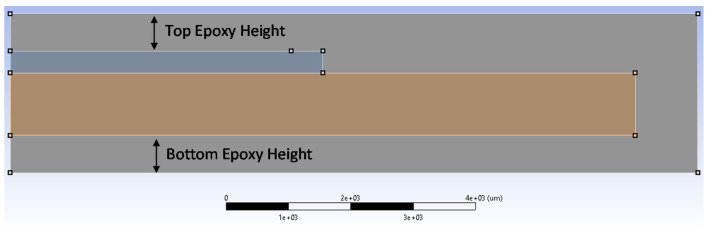
Dimension parameters for parametric analysis.

**Figure 10:**
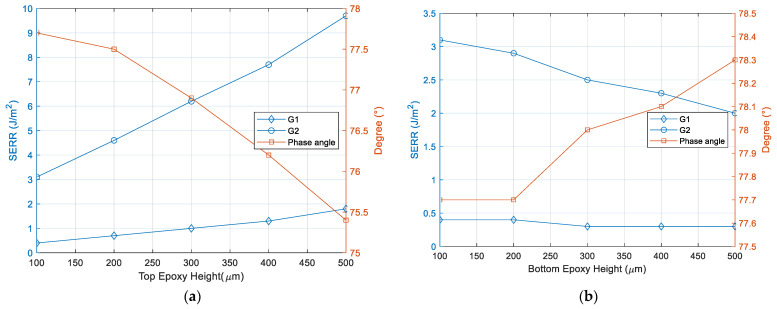
(**a**) Fracture parameters as function of top epoxy height. (**b**) Fracture parameters as function of bottom epoxy height.

**Figure 11:**
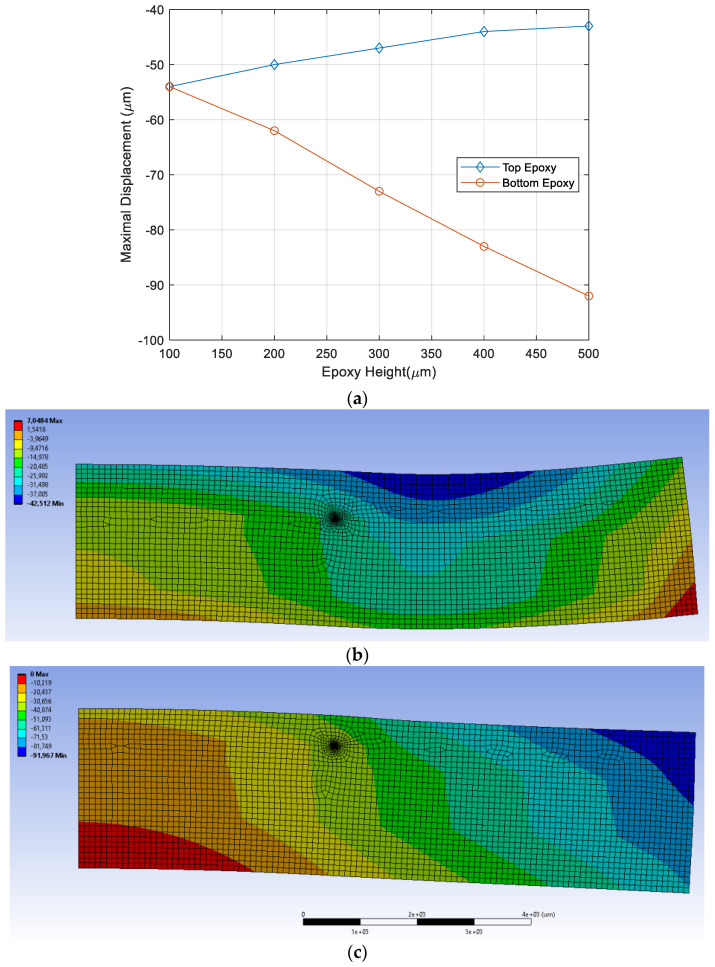
Bending mode as a function of epoxy height. (**a**) Maximal displacement vs. Epoxy height (**b**) Deformation of package when top epoxy height is 400 µm; (**c**) Deformation of package when bottom epoxy height is 500 µm.

Given with fracture analysis results, geometric parameters of the packaging have been studied to find optimal package dimension. The dimension parameters of interest in the package design are top epoxy height, bottom epoxy height as indicated in Figure 9. Initial thickness of top and bottom epoxy is 100 µm and the crack length is fixed to 0.5 mm. Figure 10 shows the thickness effect on fracture parameters, strain energy release rate (SERR) and phase angle. SERR increases with top epoxy thickness, while it reduces with bottom epoxy thickness. Closer examination on the bending mode of the package reveals that bending modes are dependent on the ratio between top and bottom epoxy height, as shown in Figure 11. When top epoxy height becomes thicker than that of bottom epoxy, opening the fracture mode becomes dominant due to the packaging bending mode. However, increasing bottom epoxy thickness maintains the initial bending mode making shear fracture mode far more dominant. Thus, it is recommended that bottom epoxy height should be always thicker than that of top epoxy as shear mode adhesion has greater than that of opening mode. It makes the Si chip under tensile stress, which reduces Si chip delamination from carrier substrate.

## 4. Conclusions

Biocompatible packaging plays a more and more important role in implantable medical devices due to emergence of new technology such as neural prosthesis. Evolution of the biocompatible packaging has been recently reported; it has been recognized that flip-chip bonding with a bare chip is one of best way of packaging in terms of miniaturization as is conventional electronics packaging. However, microfabrication-like packaging has been frequently reported as advanced UTC (Ultra-Thin Chip) technology is introduced. The major advantage of UTC technology is the capability of maximum miniaturization and thus, biocompatible packaging can take advantage of this new technology as package mechanical stress can cause undesirable FBR (Foreign Body Reaction) during implant. The biocompatible packaging approach can be categorized in two different ways, polymer encapsulation of conventional package and chip-level packaging. Material properties for encapsulation polymers and diffusion barrier have been presented in view of long-term biocompatible packaging. Diffusion barrier is a dielectric or ceramic layer to prevent liquid penetration or leaching of toxic chemical out of packaged Si chip, while encapsulating polymer is a protection polymer with lower elasticity causing smaller mechanical stress to surrounding tissue. As encapsulating polymer has multiple interfaces with the packaged objects such as sensors, Si chip, bumps, and bonding wire, there would be interfacial delamination caused by imperfect manufacturing of polymer coating, resulting in voids or microcracks. FEM modeling and simulation, based on fracture mechanics, is an efficient way to understand of the failure mode due to the interfacial delamination. At FEM modeling and simulation, phase angle based on stress intensity factor of mode I and mode II is used to find principal failure mode of encapsulation polymer for biocompatible package. Package design can be also made through FEM-based parametric study of package geometric parameters. In conclusion, this paper addresses that it is of high importance that packaging technology, material selection, modeling, and simulation are essential for long-term reliable biocompatible package.

## Figures and Tables

**Table 1:** Summarizes material properties and process conditions of polymer materials.

Properties	Polyimide +	Epoxy	Parylene-C	PDMS ++	SU8 +++
Possible thickness (µm)	3–20		1–100	10–100 with spincoating	1–300
Moisture absorption (%)	-		0.06	<1	0.55–0.65
Glass transition temperature (°C)	-	≥40 *	-	-	200–210
Thermal coefficient of expansion (ppm/K)	35	52 (below Tg) * 191 (above Tg) *	35	-	52
Tensile strength (MPa)	200	34 **	69	6.2	60
Elastic modulus (MPa)	3400	4800 **	3200	0.1–0.5	2000

+ HD microsystem HD4100 Series, * EPOTEK-302 data sheet, ** Araldite 2014, ++ Nusil MED-1000, +++ Microchem SU8-2000 and SU8-3000 series.

**Table 2:** Material properties of diffusion barrier layer of the biocompatible package *.

Material Properties	SiO_2_	Si_3_N_4_	SiC	Al_2_O_3_
Density (g/cm^3^)	2.65	3.44	3.21	3.95
Thermal coefficient of expansion (10^−5^ K^−1^)	0.05	0.28	0.44	0.70
Elastic modulus (GPa)	66.3	310	90	330
Poisson ratio	0.15	0.27	0.35	0.22
Tensile strength (MPa)	45	400	240	240

* Table 2 is partially taken from reference [21].

**Table 3:** Material properties and model dimension.

Name	Elastic Modulus (GPa)	Poisson Ratio	Coefficient of Thermal Expansion (/°C)	Dimension	
				Width (mm)	Height (µm)
Epoxy *	1.58	0.4	60.7 × 10^−6^	11	3350
Silicon **	190	0.28	3.1 × 10^−6^	5	350
PCB (FR4) ***	24	0.15	14.5 × 10^−6^	10	1000

* Material properties has been extracted from Ref. [29]. ** Material properties have been taken from Ref [56]. *** Material properties have been extracted from Ref [57].
